# Does the use of high PEEP levels prevent ventilator-induced lung injury?

**DOI:** 10.5935/0103-507X.20170032

**Published:** 2017

**Authors:** Guillermo Bugedo, Jaime Retamal, Alejandro Bruhn

**Affiliations:** 1 Departamento de Medicina Intensiva, Pontificia Universidad Catolica de Chile - Santiago, Chile.

**Keywords:** Acute respiratory distress syndrome, Ventilator-induced lung injury, Respiration, artificial

## Abstract

Overdistention and intratidal alveolar recruitment have been advocated as the main physical mechanisms responsible for ventilator-induced lung injury. Limiting tidal volume has a demonstrated survival benefit in patients with acute respiratory distress syndrome and is recognized as the cornerstone of protective ventilation. In contrast, the use of high positive end-expiratory pressure levels in clinical trials has yielded conflicting results and remains controversial. In the present review, we will discuss the benefits and limitations of the open lung approach and will discuss some recent experimental and clinical trials on the use of high versus low/moderate positive end-expiratory pressure levels. We will also distinguish dynamic (tidal volume) from static strain (positive end-expiratory pressure and mean airway pressure) and will discuss their roles in inducing ventilator-induced lung injury. High positive end-expiratory pressure strategies clearly decrease refractory hypoxemia in patients with acute respiratory distress syndrome, but they also increase static strain, which in turn may harm patients, especially those with lower levels of lung recruitability. In patients with severe respiratory failure, titrating positive end-expiratory pressure against the severity of hypoxemia, or providing it in a decremental fashion after a recruitment maneuver, is recommended. If high plateau, driving or mean airway pressures are observed, prone positioning or ultraprotective ventilation may be indicated to improve oxygenation without additional stress and strain in the lung.

## Abstract (pt)

A distensão excessiva e o recrutamento alveolar pelo volume corrente foram defendidos como os principais mecanismos físicos responsáveis pela lesão pulmonar induzida pelo ventilador. A limitação do volume corrente demonstrou benefícios quanto à sobrevivência em pacientes com síndrome da angústia respiratória aguda e é reconhecida como a pedra fundamental da ventilação protetora. Em contraste, o uso de elevados níveis de pressão positiva expiratória final em estudos clínicos gerou resultados conflitantes e ainda é um assunto controvertido. Nesta revisão, discutimos os benefícios e as limitações da abordagem de pulmão aberto, e debatemos alguns recentes estudos experimentais e clínicos, referentes ao uso de níveis baixos e moderados de pressão positiva expiratória final. Também distinguimos o estiramento dinâmico (volume corrente) do estático (pressão expiratória final positiva e pressão média nas vias aéreas) e discutimos seus papéis na indução da lesão pulmonar induzida pela ventilação. As estratégias com elevada pressão positiva expiratória final claramente diminuem a hipoxemia refratária em pacientes com síndrome da angústia respiratória aguda, porém também aumentam o estiramento estático, que, por sua vez, pode ser lesiva aos pacientes, especialmente para aqueles com nível mais baixo de recrutabilidade pulmonar. Em pacientes com insuficiência respiratória grave, recomenda-se a titulação da pressão positiva expiratória final contra a gravidade da hipoxemia, ou sua aplicação de uma forma decrescente após manobra de recrutamento. Caso sejam observadas elevadas pressões de platô, *driving pressure* ou pressão média nas vias aéreas, a posição prona ou ventilação ultraprotetora podem ser indicadas para melhora da oxigenação, sem estresse adicional e estiramento dos pulmões.

## INTRODUCTION

Over the last few decades, several experimental and clinical studies have noted the relevance of physical mechanisms in generating or perpetuating ventilator-induced lung injury (VILI).^(1)^ Overdistention due to a high tidal volume (Vt) or end inspiratory pressures, and the repeated opening and closing of distal bronchi and unstable alveoli resulting in high stress and strain, have been proposed as the main physical mechanisms responsible for VILI. The use of a low tidal volume instead of a large one led to a marked effect on survival in a large prospective, randomized, multicenter trial of patients with acute respiratory distress syndrome (ARDS), initiating the era of low tidal volume ventilation or protective ventilation.^(2)^ However, the use of high positive end-expiratory pressure (PEEP) strategies has yielded conflicting clinical outcome results.

Positive end-expiratory pressure was been used to improve hypoxemia in patients with ARDS shortly after the first description of the syndrome.^(3)^ Later, higher levels of PEEP along with recruitment maneuvers were proposed to prevent intratidal alveolar recruitment and improve survival. However, despite several translational and clinical studies, the effectiveness of these maneuvers remains controversial.

In the present article, we will present a short historical review on the use of high PEEP levels in patients with ARDS and will discuss some recent experimental and clinical trials in different clinical settings. In our view, the benefit from protective ventilation is mainly due to a decrease in stress and strain secondary to the use of a low tidal volume, and hence cyclic strain, in a highly heterogeneous lung. In contrast, the protective effect of PEEP on VILI is more debatable, as although it is highly effective at improving oxygenation, it may also increase strain and stress on the lung.

### Lung injury at low lung volumes and the open lung approach

Ventilation that occurs at low lung volumes can cause injury through multiple mechanisms, including the repetitive opening and closing of airways and lung units, effects on surfactant function, and regional hypoxia.^(1)^ Different experimental models have shown that the repetitive tidal recruitment and derecruitment (R/D) of small airways does occur at low or absent PEEP levels, promoting or increasing markers of VILI, while recruitment maneuvers and high PEEP levels result in improved oxygenation and less histological damage.

These observations are supported by two clinical trials using an open lung approach with high PEEP levels and low tidal volumes. These studies found positive results for this method when compared against a "conventional" strategy consisting of low to moderate PEEP and large tidal volumes.^(4,5)^ The effect of PEEP in these studies should be assessed carefully, as tidal volume limitation in the open lung strategy could be responsible for the observed benefit.

The concept of "baby lung" and a pioneering study by Hickling on permissive hypercapnia^(6)^ led several groups to conduct prospective studies that compared a tidal volume and/or pressure limitation strategy against a more conventional approach (Table 1).^(2,4,5,7-9)^ The largest and most important of these studies showed that the use of a tidal volume of 6mL/kg IBW reduced mortality by approximately 25% compared with ventilation with 12mL/kg IBW in over 800 patients with ARDS.^(2)^

**Table 1:** Ventilatory parameters at 24 hours and mortality in clinical studies comparing a protective strategy, tidal volume (Vt) limitation, versus a control group (top panel); a strategy of high positive end-expiratory pressure versus low positive end-expiratory pressure or minimal distension (middle panel); and a conventional protective strategy versus high frequency oscillatory ventilation (HFOV) (lower panel) in patients with acute respiratory distress syndrome. The driving pressure of the respiratory system (ΔP) is calculated as the difference between the plateau pressure and positive end-expiratory pressure. Note that a larger difference of driving pressure between groups (Dif ΔP) is associated with differences in mortality

Author	Year	N	Vt	P_pl_	PEEP	ΔP	Mortality %	Vt	P_pl_	PEEP	ΔP	Mortality %	Dif ΔP	p value†
			**Protective strategy**					**Control group**						
Brochard et al.^(7)^	1998	108	7.1	25.7	10.7	15	46.6	10.3	31.7	10.7	21	37.9	6	ns
Stewart et al.^(8)^	1998	120	7.2	22.3	8.6	13.7	48.0	10.8	26.8	7.2	19.6	46.0	5.9	ns
Ranieri et al.*^(5)^	1999	44	7.6	24.6	14.8 *	9.8	38.0	11.1	31	6.5	24.5	58.0	14.7	0.19
Brower et al.^(9)^	1999	52	7.3	27	9.3	17.7	50.0	10.2	30	8.2	21.8	46.0	4.1	ns
Amato et al.*^(4)^	1998	53	6	31.8	16.3 *	15.5	38.0	12	34.4	6.9	27.5	71.0	12	< 0.001
ARDSnet^(2)^	2000	861	6.1	25	9.4	15.6	31.0	11.9	33	8.6	24.4	39.8	8.8	0.007
			**High PEEP**					**Low PEEP**						
ALVEOLI^(10)^	2004	549	6.1	27	14.7	12.3	27.5	6.0	24	9.1	14.9	24.9	2.6	ns
Mercat et al.^(12)^	2008	767	6.1	27.5	15.8	11.7	35.4	6.1	21.1	8.4	12.7	39.0	1.0	ns
Meade et al.^(11)^	2008	983	6.8	30.2	15.6	14.6	36.4	6.8	24.9	10.1	14.8	40.4	0.2	ns
Kacmarek et al.^(15)^	2016	200	5.6	27.9	15.8	11.8	22	6.2	25.2	11.6	13.8	27	2.0	0.18
			**Conventional protective**					**HFOV**						
Young et al.^(31)^	2013	795	8.3	30.9	11.4	19.5	41.1	-	-	-	-	41.7	-	ns
Ferguson et al.^(32)^	2013	548	6.4	29.0	15.0	14.0	35.0	-	-	-	-	47.0	-	0.005

PEEP - positive end-expiratory pressure; Vt - tidal volume; P_pl_ - plateau pressure; ΔP - driving pressure; Dif ΔP - difference of driving pressure; HFOV - high frequency oscillatory ventilation; ns - not significant.

* Ranieri and Amato studies also use high PEEP in the protective strategy.

† The p value refers to the differences in mortality between groups.

### High PEEP strategies after the ARDSnet low tidal volume trial

After the ARDSnet low Vt study, three large randomized trials compared high and moderate PEEP strategies using low tidal volumes in both groups (Table 1).^(10-12)^ None of these studies showed differences in mortality. However, a meta-analysis of these three studies suggested a small survival benefit from the high PEEP strategy in the subgroup of patients with a ratio arterial oxygen partial pressure to fractional inspired oxygen (PaO_2_:FiO_2_) < 200.^(13)^ Considering only the studies from Meade et al.^(8)^ and Mercat et al.,^(9)^ which defined refractory hypoxemia *a priori*, high PEEP strategies led to significantly fewer episodes of refractory hypoxemia and required fewer rescue therapies.^(14)^

A recent trial comparing an open lung approach (OLA study) with the ARDSnet study involved 200 patients with a PaO_2_:FiO_2_ ratio < 200 after a period of stabilization of at least 12 hours of protective ventilation, thus selecting a group with higher disease severity (Table 1).^(15)^ This study had a low power to detect any relevant effect on mortality, but showed improved oxygenation and, more importantly, lower driving pressures, which may translate into lower dynamic strain (vide infra).^(16,17)^

A large randomized trial (ART) led by Brazilian investigators is assessing the effects of alveolar recruitment followed by decremental PEEP titration to optimize static compliance. This trial involving 1,100 patients is expected to be completed in 2017 and will provide important information on the effect of the open lung approach for patients with ARDS.^(18)^

### Why were all these studies negative?

The use of PEEP makes sense for two reasons: first, by recruiting unstable alveoli, PEEP improves gas exchange and tissue oxygenation; second, PEEP reduces and redistributes the heterogeneous mechanical stresses of tidal ventilation.^(19)^ Only the first assumption has proven to be true in patients, as the mechanical response to PEEP is highly variable in patients with ARDS.^(20)^

Animal experiments showing the benefits of high PEEP strategies usually use a highly recruitable model of lung damage, which does not necessarily translate to human ARDS.^(21,22)^ In contrast, most clinical trials in patients with ARDS have not assessed their recruitability (Table 1). Thus, the benefit of a high PEEP strategy in patients with severe ARDS and refractory hypoxemia may be obscured by the induction of overdistention and further lung injury in patients with less severe forms of respiratory failure, and thus less recruitable lungs.

An example of this lower level of recruitability occurs in the perioperative setting.^(23)^ A large clinical trial using a high level of PEEP (12cmH_2_O) and recruitment maneuvers during open abdominal surgery showed no protection against postoperative pulmonary complications.^(24)^ In contrast, in 400 patients undergoing major abdominal surgery and at high risk of pulmonary complications, a strategy using a low tidal volume and moderate levels of PEEP decreased major pulmonary and extrapulmonary complications within the first 7 days, compared to a conventional strategy (Vt 10 - 12mL/kg IBW and no PEEP).^(25)^

### Global strain and cyclic strain

In a recent experimental model, Protti et al. demonstrated that a lung strain (the ratio between tidal volume and functional residual capacity) greater than 1.5 - 2 was necessary to induce lung damage in pigs without previous lung injury.^(26)^ In a second experiment, Protti et al. used several combinations of tidal volume (dynamic strain) and PEEP (static strain) to induce a similar level of global strain (the sum of static and dynamic strain) large enough to induce lung injury.^(16)^ Dynamic strain, also called cyclic strain, is mainly determined by tidal volume, while static strain represents the volume of gas caused by PEEP and may be well represented by mean airway pressure.^(27)^ A ventilatory strategy consisting of small dynamic (lower Vt) and large static (higher PEEP) strains decreased several markers of lung injury and mortality, suggesting that static strain is less harmful than dynamic strain.

In humans with ARDS, Caironi et al. showed that high PEEP levels decreased R/D only in patients with highly recruitable lungs, whereas no differences were observed in patients with lower levels of recruitability.^(28)^ However, strain increased with higher PEEP levels independent of lung recruitability. In a small set of patients with ARDS, we showed that global strain increased along PEEP levels and plateaued at airway pressure.^(29)^ More recently, increasing PEEP from 9 to 15cmH_2_O along with low Vt ventilation did not decrease tidal R/D but consistently increased tidal recruitment and hyperinflation.^(30)^

### Lessons from high-frequency oscillatory ventilation clinical trials

**Figure 1:**
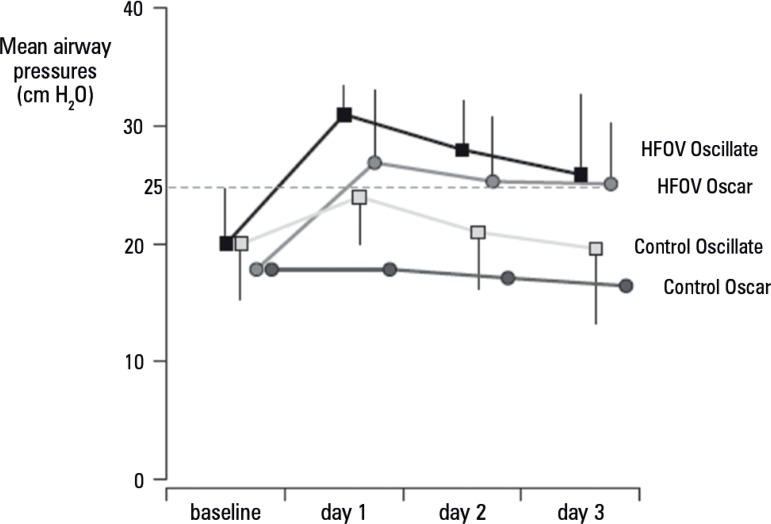
Mean airway pressures in Oscillate (squares) and Oscar (circles) studies. Data are from tables 3S and 4S (Oscillate) and from table 2 (Oscar). In the Oscar trial, mean airway pressures in the control arm were not given and were calculated as P_mean_=PEEP + 1/3(Δ P_plateau_-PEEP), considering an inspiratory time from 1:2. HFOV - high-frequency oscillatory ventilation.

High-frequency oscillatory ventilation (HFOV), by allowing greater end-expiratory lung volume while minimizing cyclic strain, resembles a high PEEP low Vt strategy, which seems ideal for lung protection in patients with ARDS. However, two recent multicenter, randomized trials did not show a survival benefit to this strategy, and in one study HFOV led to more deaths than a conventional approach (Table 1).^(31,32)^ Mean airway pressure (Paw) in both HFOV arms was higher (above 25cmH_2_O, Figure 1) than that of controls, which could reflect a higher global strain.^(16)^ As cyclic strain is minimized by HFOV (due to a much lower tidal volume), the higher global strain may only be a result of the higher static strain. The greater levels of vasopressor and intravenous fluid administration in the Oscillate trial, induced by a higher Paw, may help support this hypothesis.

In summary, in patients with moderate to severe ARDS, the higher global strain observed with HFOV may explain its lack of benefit -or even its harm- as found in recent trials, and may suggest a limit for PEEP titration. As high PEEP levels increase mean airway pressure, and hence static and global strain, Paw values above 25cmH_2_O may suggest a limit when a more conservative prone or ultraprotective approach should be used.

### Moving to ultraprotective ventilation

In contrast to the controversial data on PEEP, limiting tidal volume has been shown to be beneficial, leading to fewer complications and/or less mortality in different groups of patients with mechanical ventilation and becoming the standard for ventilation in critically ill patients.^(2,25,33)^ The negative results in recent trials of high versus low/moderate PEEP have been ascribed to the use of low Vt in both arms (along with moderate PEEP in controls), precluding the trigger for injurious ventilation.

Recent data suggest that inhomogeneity in human ARDS acts to increase stress and is associated with disease severity and mortality.^(21,34)^ In an experimental model in pigs, applying very high stress and strain to the lung parenchyma leads to abnormal lung densities that are detected within 8 hours of ventilation at inhomogeneous interfaces and increase exponentially until lung edema develops after 20 hours.^(35)^

Independent of lung inhomogeneity and recruitability, tidal volume limitation will always suppress the main physical mechanisms involved in VILI. Using dynamic CT in nine patients with ARDS, lowering Vt from 12 to 6mL/kg IBW was found to not only decrease transpulmonary pressure and hyperinflation but also diminish the cyclic R/D of unstable alveoli.^(36)^

In a clinical setting, a small study of 10 patients with ARDS and plateau pressures of 28 - 30cmH_2_O despite a Vt of 6mL/kg IBW, a further decrease in Vt to 4mL/kg IBW and partial extracorporeal carbon dioxide removal reduced pulmonary cytokine concentrations after 72 hours.^(37)^ The use of a Vt of 3mL/kg IBW along with extracorporeal CO_2_ removal may have benefited patients with PaO_2_:FiO_2_ ratios < 150, when compared with a Vt 6mL/kg IBW protective strategy.^(38)^ Using dynamic CT, we showed that the reduction in Vt from 6 to 4mL/kg IBW decreased R/D, while partial pressure of carbon dioxide (PaCO_2_) and pH could be maintained at clinical levels if instrumental dead space was minimized.^(39)^

New evidence on protective ventilation in ARDS patients suggests that paralysis and prone positioning also have a major role in improving clinical outcomes.^(40,41)^ The striking data from these studies contrast with those comparing higher and lower PEEP settings. In particular, prone positioning may enhance the effects of high PEEP by preventing the negative effects of PEEP on tidal hyperinflation.^(42)^

**Figure 2:**
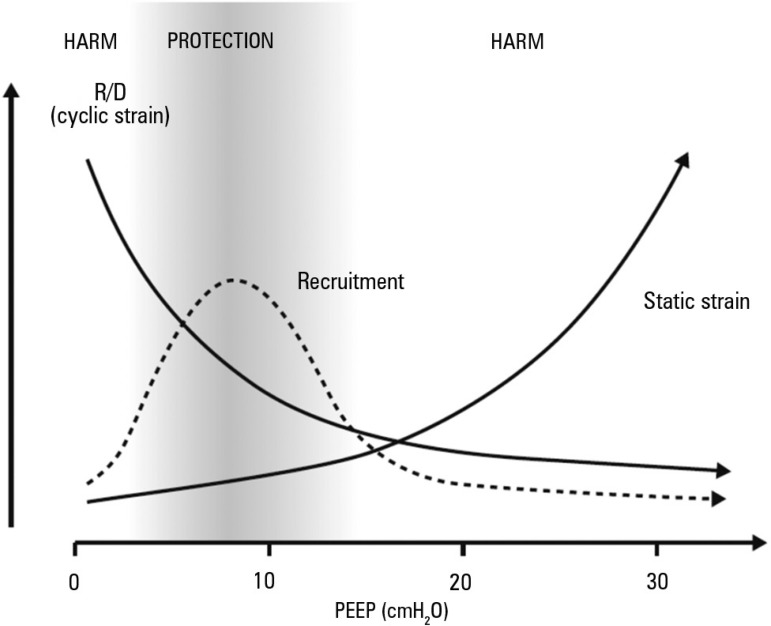
Effect of increasing levels of positive end-expiratory pressure on alveolar recruitment, tidal recruitment and derecruitment and static strain. From zero end-expiratory pressure to a positive end-expiratory pressure of 5cmH_2_O, there was marked recruitment and a decrease in recruitment and derecruitment, which provided a protective effect. Positive end-expiratory pressure levels above 15cmH_2_O should be carefully titrated, as the impact on recruitment is less evident and strain may increase.

Summarizing these data, we suggest that the mechanical benefit of PEEP is most often found in patients with acute respiratory failure from 5 to 12 or 15cmH_2_O, as alveolar recruitment prevails and oxygenation improves (Figure 2). At these PEEP levels, recruiting collapsed alveoli may also reduce driving pressure (dynamic strain), which could translate into less VILI.^(15)^ However, although there is no clear limit, the use of high PEEP levels above 12 or 15cmH_2_O) should be carefully titrated, as higher static strain and overdistention may prevail over recruitment.^(28-30)^

**Figure 3:**
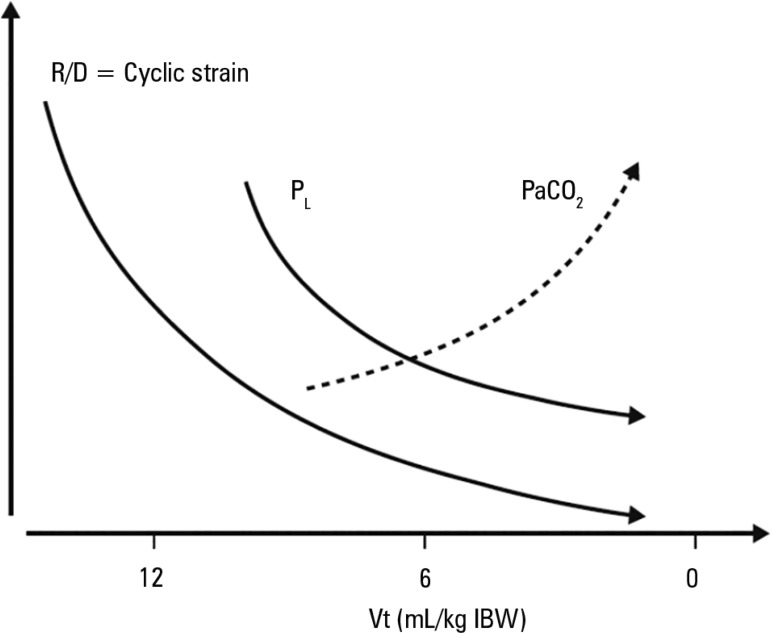
Effect of different tidal volumes on tidal recruitment and derecruitment, partial pressure of carbon dioxide levels and transpulmonary pressure. A decrease in tidal volume will always induce a decrease in transpulmonary pressure, but a very low tidal volume may increase partial pressure of carbon dioxide and decrease pH. Vt - tidal volume; R/D - tidal recruitment and derecruitment; PaCO_2_ - partial pressure of carbon dioxide; P_L_ - transpulmonary pressure.

In contrast, a decrease in tidal volume below physiological levels of 3 to 4mL/kg IBW will always confer the benefit of lower transpulmonary pressure, which is the main determinant of cyclic strain. Theoretically, a Vt of 0 should eliminate the cyclic R/D of unstable alveoli, but is accompanied by the constraints of hypercapnia and respiratory acidosis (Figure 3). This is the principle behind ultraprotective ventilation and extracorporeal membrane oxygenation. However, the role of these methods in severe respiratory failure has yet to be demonstrated.

## FINAL COMMENTS

We strongly support the use of an open lung approach in patients with severe acute respiratory distress syndrome, as it decreases refractory hypoxemia.^(13-15)^ However, whether high levels of positive end-expiratory pressure prevent ventilator induced lung injury is still controversial. The clinical evidence suggests that tidal volume limitation is the cornerstone of protective ventilation. Thus, the proven benefit of high positive end-expiratory pressure strategies in decreasing refractory hypoxemia should be carefully weighed against the induction of added strain and overdistention, as it may be harmful under certain clinical conditions, such as in perioperative patients, patients with mild respiratory failure or patients with interstitial diseases.

Limiting tidal volume (and thus cyclic strain) and applying moderate positive end-expiratory pressure levels (between 8 to 12cmH_2_O) to prevent excessive stress and strain on the lung may be sufficient for most ventilated patients. In patients with severe respiratory failure, titrating positive end-expiratory pressure against the severity of hypoxemia or in a decremental fashion to obtain better compliance or driving pressure is recommended.^(15,17)^ When plateau pressures are above 30 - 35cmH_2_O, driving pressures are above 15 - 20cmH_2_O or mean airway pressures are above 25cmH_2_O, the adoption of prone positioning or ultraprotective ventilation may be indicated to improve oxygenation without inducing added stress and strain on the lung.
